# Clinical Evaluation of a Combined Deep Learning–Reconstructed Readout-Segmented Echo-Planar Imaging and Water-Excitation Spectral Fat-Saturation Protocol for Breast Diffusion-Weighted Imaging at 3T Breast MRI

**DOI:** 10.3390/diagnostics16131958

**Published:** 2026-06-24

**Authors:** Jung Min Choi, Soyeoun Lim, Eun Jung Choi, MunYoung Paek, Wei Liu, Minseo Bang, Jung Hee Byon

**Affiliations:** 1 Department of Radiology, Ulsan University Hospital, University of Ulsan College of Medicine, Ulsan 44033, Republic of Korea; choi22391@naver.com (J.M.C.); soyeoun.lim.xr@uuh.ulsan.kr (S.L.); 2 Department of Radiology, Jeonbuk National University Medical School, Jeonju 54907, Republic of Korea; cejcej80@hanmail.net; 3 Research Institute of Clinical Medicine, Jeonbuk National University–Biomedical Research Institute, Jeonbuk National University Hospital, Jeonju 54907, Republic of Korea; 4 Siemens Healthineers Ltd., Seoul 06620, Republic of Korea; munyoung.paek@siemens-healthineers.com; 5 Siemens Healthineers AG, 91052 Erlangen, Germany; weiwlliu@siemens-healthineers.com

**Keywords:** diffusion magnetic resonance imaging, echo-planar imaging, deep learning, fat suppression, breast neoplasms

## Abstract

**Objectives**: This study evaluates the protocol-level image quality and quantitative diffusion metrics of a clinically implemented deep-learning–reconstructed readout-segmented echo-planar imaging protocol with water-excitation spectral fat saturation (DL-rs-EPI with WEXfs) compared with conventional rs-EPI using spectral attenuated inversion recovery (SPAIR) at 3 T. **Methods**: Overall, 80 patients underwent breast magnetic resonance imaging (MRI) with both conventional rs-EPI with SPAIR and DL-rs-EPI with WEXfs protocols (b-values: 0, 800, and 1200 s/mm^2^). ROI-based relative image-quality metrics, including signal-to-noise ratio (SNR), contrast-to-noise ratio (CNR), and lesion contrast, were assessed at b = 800 and b = 1200 s/mm^2^; apparent diffusion coefficient (ADC) values were calculated using multi-b-value data. Fat suppression, background diffusion signal, lesion conspicuity, and artifact severity were qualitatively evaluated. A temperature-controlled diffusion phantom (CaliberMRI) was scanned; ADC values were compared with reference values at 24 °C. **Results**: DL-rs-EPI with WEXfs demonstrated higher ROI-based relative SNR estimates (b800: 5.79 vs. 5.28; b1200: 5.41 vs. 4.94; *p* < 0.001) and CNR estimates (b800: 3.35 vs. 3.12, *p* = 0.024; b1200: 3.67 vs. 3.37, *p* = 0.001), with unchanged lesion contrast. Tumor ADC values were comparable between protocols, whereas normal fibroglandular tissue ADC values were slightly higher, and ADC contrast increased with DL-rs-EPI with WEXfs. Phantom ADC values from both protocols closely matched reference values at 24 °C, without significant differences. DL-rs-EPI with WEXfs demonstrated more homogeneous fat suppression and reduced background diffusion signal, with comparable lesion conspicuity and artifact severity. **Conclusions**: The combined DL-rs-EPI with WEXfs protocol demonstrated improved qualitative and relative quantitative image quality while preserving tumor ADC measurements. As a protocol-level evaluation, these composite improvements support its clinical feasibility for high-quality breast DWI without implying the isolated effect of DL reconstruction alone.

## 1. Introduction

Breast cancer remains highly prevalent worldwide, contributing to the increasing use of breast magnetic resonance imaging (MRI) [1,2,3]. Dynamic contrast-enhanced (DCE) T1-weighted imaging (T1WI) remains the cornerstone of breast MRI protocols [4]. In parallel, diffusion-weighted imaging (DWI) has increasingly been incorporated into multiparametric breast MRI as a complementary technique that provides functional information without contrast agents [5]. Consequently, interest in non-contrast breast MRI, particularly DWI, has continued to grow [5].

Contemporary consensus statements describe breast DWI as commonly performed using echo-planar imaging (EPI)-based readout strategies [6,7]. Single-shot EPI (ss-EPI) acquires all k-space lines for a given slice within a single excitation, enabling rapid image acquisition (50–100 ms per slice) and reducing sensitivity to patient motion [7]. However, the long echo train inherent to ss-EPI makes it susceptible to T2* decay and magnetic field inhomogeneity, leading to geometric distortion, image blurring, and limited spatial resolution [5,7]. To overcome these limitations, multi-shot EPI techniques have been introduced by segmenting k-space acquisition across multiple excitations. Multi-shot EPI techniques, such as readout-segmented EPI (rs-EPI), divide k-space acquisition into multiple segments along the readout direction, thereby shortening the echo train length and the echo spacing. This approach reduces susceptibility-induced geometric distortion and image blurring, enabling higher spatial resolution compared with ss-EPI [5,7].

Deep learning (DL)-based reconstruction has recently been introduced as a vendor-implemented approach that may improve perceived image quality in diffusion MRI by reducing apparent noise and reconstruction-related artifacts [8,9]. Recent reviews have emphasized the expanding role of DWI and accelerated MRI techniques in breast imaging, particularly in the context of non-contrast and abbreviated MRI protocols [10]. Previous studies have investigated DL reconstruction mainly in ss-EPI-based breast DWI [8,9] or in alternative multi-shot diffusion techniques [11,12], with reported improvements in image quality while preserving ADC measurements. However, evidence regarding the combined use of DL reconstruction with rs-EPI and alternative fat-suppression strategies remains limited. Therefore, further protocol-level evaluation is needed to determine whether a clinically implemented DL-rs-EPI protocol can improve image quality while preserving quantitative diffusion measurements in high-resolution breast DWI.

Another key technical consideration in breast DWI is fat suppression. Because low apparent diffusion coefficient (ADC) values are an important imaging feature suggestive of malignancy, fat contamination can spuriously reduce ADC measurements and increase false-positive findings. Accordingly, several organizations—including the European Society of Breast Imaging [6], the Quantitative Imaging Biomarkers Alliance/Radiological Society of North America [13], and the Korean Radiological Society [7]—recommend spectral attenuated inversion recovery (SPAIR) as the preferred fat-suppression technique for breast DWI. SPAIR is more robust to B1 inhomogeneity than spectral fat saturation and provides a higher signal-to-noise ratio (SNR) than short inversion time inversion recovery. However, a recent report suggested that combining water excitation (WEX), which selectively excites water protons, with spectral fat saturation may provide superior image quality compared with SPAIR in breast DWI by improving robustness to B1 inhomogeneity [14]. Notably, this comparison was performed in ss-EPI-based breast DWI rather than rs-EPI, and evidence specific to rs-EPI breast DWI remains limited.

Therefore, this study aimed to evaluate the protocol-level image quality and quantitative diffusion metrics of a clinically available DL-rs-EPI DWI protocol with water-excitation spectral fat saturation compared with a conventional rs-EPI protocol with SPAIR at 3 T breast MRI. We assessed qualitative image quality, ROI-based SNR and CNR estimates, lesion contrast, and ADC measurements in patients with pathologically confirmed breast cancer, and performed phantom validation to examine the stability of ADC measurements under controlled conditions.

## 2. Materials and Methods

### 2.1. Study Design and Patients

This single-center, retrospective study was conducted at Ulsan University Hospital. The Institutional Review Board (IRB) approved this study, and the requirement for informed consent was waived (IRB No. 2025-09-022). Between 22 July 2024 and 10 February 2025, a total of 326 patients underwent breast MRI for breast cancer diagnosis. Patients were eligible for inclusion if they (i) had pathologically confirmed breast cancer and (ii) underwent breast MRI in a single session, including both conventional rs-EPI and DL-rs-EPI with WEXfs. Patients were excluded if they met any of the following criteria: (i) neoadjuvant chemotherapy before MRI; (ii) excisional biopsy performed before the MRI; (iii) absence of a clearly visible index breast malignancy on MRI, precluding qualitative scoring; (iv) absence or insufficiency of normal breast parenchyma for standardized comparisons due to a history of contralateral mastectomy or male breast cancer; (v) technical failure or acquisition error affecting either the conventional rs-EPI or DL-rs-EPI with WEXfs sequences; or (vi) incomplete clinical or pathological data.

### 2.2. MRI Acquisition

All examinations were performed using a 3-T MR scanner (MAGNETOM Vida; Siemens Healthineers, Forchheim, Germany) with a dedicated 18-channel phased-array breast coil, with patients in the prone position. The breast MRI protocol included fat-saturated T2-weighted turbo spin-echo imaging and two DWI sequences (conventional rs-EPI and DL-rs-EPI with WEXfs), followed by five phases of DCE T1WI after intravenous administration of a gadolinium-based contrast agent (gadoterate meglumine, Dotarem; Guerbet, Paris, France). The acquisition parameters of the two DWI sequences are summarized in Table 1.

### 2.3. DL-Based MRI Reconstruction

DL-rs-EPI images were reconstructed using a DL reconstruction framework (research prototype; Siemens Healthineers, Forchheim, Germany) integrated into a prototype rs-EPI diffusion sequence. DL reconstruction was performed using an inline raw-data-to-image reconstruction algorithm based on a variational network framework. The algorithm reconstructs undersampled multi-coil MRI data by combining learned regularization with data-consistency constraints, thereby reducing noise and residual aliasing while preserving consistency with the acquired k-space data [15]. The network was based on a variational network architecture with 17 unrolled iterations [16,17] and operated directly on raw k-space data together with pre-calculated coil sensitivity maps, which were estimated using vendor-provided autocalibration data. In the vendor-provided implementation, the model had been trained in a supervised manner using a large DWI dataset acquired on 1.5-T and 3-T MR systems (MAGNETOM, Siemens Healthineers, Forchheim, Germany), and the final network was implemented for inline reconstruction on the scanner. The DL reconstruction framework with WEXfs, which has previously been demonstrated for abdominal DWI [18], was adapted for rs-EPI by incorporating dedicated phase-correction and segment-combination steps, ensuring compatibility with the rs-EPI sequence [19].

Importantly, the present study did not develop, train, or fine-tune a new DL model. Instead, it clinically evaluated a vendor-provided inline DL reconstruction framework implemented on the MRI system. Therefore, no additional offline reconstruction, model training, or user-defined hyperparameter optimization was performed by the authors. The same reconstruction configuration was applied consistently to all included examinations. Detailed internal parameters, including the full network architecture, loss function, detailed training procedure, and tunable hyperparameters, were proprietary to the vendor and were not available to the authors.

### 2.4. Fat Suppression Technique

For EPI-based DWI, reliable fat suppression is essential to minimize chemical shift-related ghosting and associated image degradation. In the conventional rs-EPI DWI protocol, SPAIR was employed for fat suppression due to its robustness to B0 and B1 inhomogeneities. In the prototype DL-rs-EPI DWI protocol, WEXfs was applied. In this implementation, the conventional adiabatic inversion pulse used in SPAIR was omitted, and a spectrally selective sinc-shaped fat-saturation radiofrequency pulse was utilized instead. In addition, the excitation module was replaced with a WEX sequence employing a monopolar 1-2-2-1 composite scheme designed to preferentially excite water while minimizing fat excitation, thereby improving the robustness of fat suppression. The WEXfs configuration was integrated into the rs-EPI diffusion acquisition and was compatible with simultaneous multi-slice acceleration.

### 2.5. Qualitative Image Analysis

Two dedicated breast radiologists (with 8 and 10 years of experience in breast MRI interpretation) independently performed qualitative image assessments while blinded to the DWI sequence type and reconstruction method. For each examination, overall image quality was graded using predefined ordinal scales. Homogeneity of fat suppression was rated on a four-point scale (1 = failure of fat suppression, 2 = regional failure but still interpretable, 3 = minimal failure limited to the image periphery, 4 = homogeneous fat suppression); background diffusion signal was scored on a 1–4 scale (1 = minimal, 2 = mild, 3 = moderate, 4 = marked); and lesion conspicuity was scored on a 1–3 scale (1 = poor, 2 = moderate, 3 = excellent). Furthermore, artifact severity was recorded on a 0–3 scale (0 = none, 1 = mild, 2 = moderate, 3 = severe), considering susceptibility-related distortion, aliasing, and misregistration artifacts, with emphasis on their overall impact on image interpretability. Qualitative parameters were assessed at b-values of 800 and 1200 s/mm^2^.

Qualitative scoring of global image quality parameters (fat suppression and background diffusion signal) was performed on a per-patient basis. Lesion conspicuity was evaluated on a lesion-by-lesion basis. In cases with multiple lesions, the index lesion, defined as the largest lesion with a solid enhancing component, was selected for analysis. Scores were recorded separately for each DWI sequence (DL-rs-EPI with WEXfs and rs-EPI), and inter-reader agreement was subsequently quantified.

### 2.6. Quantitative Image Analysis

Quantitative measurements were performed for each DWI reconstruction (conventional rs-EPI and DL-rs-EPI with WEXfs) using PACS software (INFINITT PACS G7, version 7.0.0.6; INFINITT Healthcare Co., Ltd., Seoul, Republic of Korea). One radiologist performed all measurements twice at each location, and the mean value was used for analysis. For paired comparison, regions of interest (ROIs) were initially delineated on one sequence and subsequently transferred to the corresponding anatomical location on the other sequence.

For each sequence, two manual freehand ROIs were drawn on the high-b-value images to encompass (i) the largest solid component of the index tumor and (ii) normal fibroglandular tissue (FGT). ROIs were consistently placed at matched anatomical locations across both reconstructions and at b-values of 800 and 1200 s/mm^2^. Normal FGT was sampled in the contralateral breast in an area without enhancement on DCE images. Tumor ROIs were delineated along lesion margins while avoiding necrotic or hemorrhagic components, with reference to T2-weighted and DCE images. For non-mass enhancement, ROIs were delineated to follow the enhancing area on DCE images to minimize inclusion of normal FGT. ROI size was maximized within lesion boundaries as far as possible to reduce sampling variability.

For each b-value (800 and 1200 s/mm^2^), SNR (SI_tumor/σ_tumor), contrast-to-noise ratio (CNR = |SI_tumor − SI_normal|/√(σ_tumor^2^ + σ_normal^2^)), and lesion contrast (Lesion contrast = SI_tumor/SI_normal) were calculated. Definitions and equations for these metrics were based on a previously published study [9]. Because DL-based reconstruction may alter image noise texture and spatial noise distribution through nonlinear denoising, the ROI-based SNR and CNR estimates were interpreted as relative image-quality indicators rather than absolute physical noise measurements. Each tumor ROI drawn on the high b-value images was transferred to the co-registered ADC map to obtain tumor ADC values. Normal tissue ADC was measured using the corresponding FGT ROI. ADC contrast was defined a priori as the difference between normal tissue ADC and tumor ADC (ADC_normal − ADC_tumor), expressed in ×10^−3^ mm^2^/s.

### 2.7. Phantom Experiment Imaging Protocol

A calibrated diffusion phantom (Diffusion Standard Model 128, CaliberMRI, Boulder, CO, USA) was used to assess the accuracy of ADC measurements obtained with the two DWI protocols. Each protocol was acquired using the same imaging parameters as in the patient study. The phantom comprised a spherical shell containing 13 vials (30 mL each) filled with traceable aqueous polyvinylpyrrolidone (PVP) solutions, with concentrations ranging from 0% (water reference) to 50%. A built-in ten-element liquid-crystal thermometer was used for MR-readable temperature monitoring, enabling temperature compensation over a range of 15–24 °C, for which manufacturer-supplied temperature-dependent reference ADC values were available.

Immediately before and after the DWI acquisitions, a three-dimensional Volumetric Interpolated Breath-hold Examination (VIBE) sequence with 1 mm isotropic resolution was acquired to document the phantom temperature and assess potential temperature drift during imaging. For each protocol and PVP vial, one radiologist placed circular ROIs on the PACS workstation to measure ADC values. Measurements were performed twice and averaged for analysis. Protocol comparisons were performed against the temperature-matched reference ADC values for each vial.

### 2.8. Statistical Analysis

Qualitative scores from the two readers were summarized as mean ± standard deviation (SD) and compared between techniques using the Wilcoxon signed-rank test (two-sided, α = 0.05). Inter-reader agreement for qualitative ordinal ratings was assessed using weighted kappa (κ) statistics with 95% confidence intervals (CIs). The κ values were interpreted as follows: 0.00–0.20, poor agreement; 0.21–0.40, fair agreement; 0.41–0.60, moderate agreement; 0.61–0.80, good agreement; and 0.81–1.00, excellent agreement. Quantitative metrics, including SNR, CNR, lesion contrast, ADC values, and ADC contrast, were summarized as mean ± SD and compared using paired *t*-tests (two-sided, α = 0.05) after verification of normality of within-pair differences; where this assumption was violated, the Wilcoxon signed-rank test was performed. Unadjusted *p*-values are presented for primary paired comparisons unless otherwise specified. Effect sizes for paired comparisons were calculated using Cohen’s dz, defined as the mean of the within-patient difference divided by the standard deviation of the within-patient difference. Differences were calculated as values from the DL-rs-EPI with WEXfs protocol minus those from the conventional rs-EPI with SPAIR protocol. Cohen’s dz values of 0.2, 0.5, and 0.8 were interpreted as small, medium, and large effect sizes, respectively. All statistical analyses were performed using SPSS version 20.0 (IBM Corp., Armonk, NY, USA).

## 3. Results

### 3.1. Characteristics of Patients and Lesions

**Figure 1:**
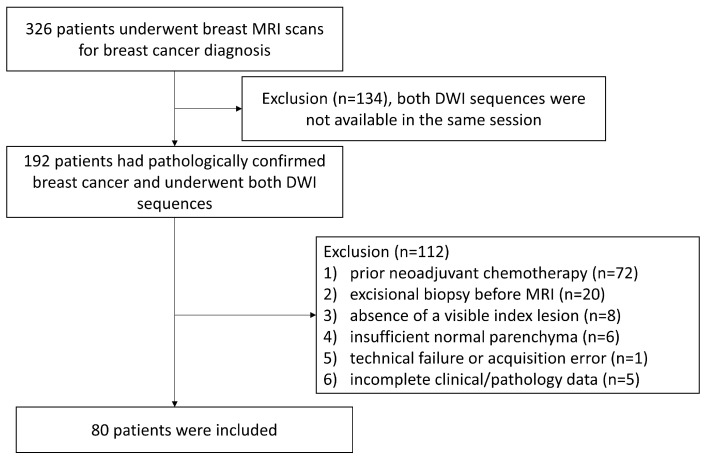
Flow diagram of study sample selection.

Among 326 patients, 192 had pathologically confirmed breast cancer and were examined using both DWI sequences. Patients were excluded for any of the following: (i) prior neoadjuvant chemotherapy (*n* = 72); (ii) excisional biopsy before MRI (*n* = 20); (iii) absence of a clearly visible index breast malignancy (*n* = 8); (iv) insufficient normal breast parenchyma (*n* = 6); (v) technical failure or acquisition error (*n* = 1); (vi) incomplete clinical or pathological data (*n* = 5). Consequently, 80 patients were included in this study (Figure 1).

The mean age of the 80 women was 55 ± 11 years (range, 25–82 years). In terms of pathology, invasive ductal carcinoma (IDC) was the most common diagnosis (55/80, 68.8%), followed by ductal carcinoma in situ (16/80, 20.0%) and other specific invasive carcinomas (9/80, 11.3%), which included invasive lobular carcinoma (ILC) (*n* = 5), invasive carcinoma with neuroendocrine differentiation (*n* = 1), invasive carcinoma with a micropapillary component (*n* = 1), mixed IDC and ILC (*n* = 1), and mucinous carcinoma (*n* = 1). Detailed information on participants and lesion characteristics is presented in Table 2.

### 3.2. Phantom ADC Measurement Results

**Figure 2:**
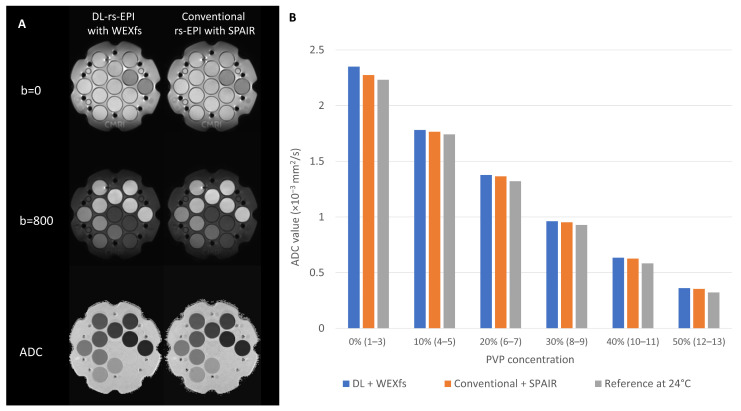
Results of the phantom experiment. (**A**) Representative diffusion-weighted images at b = 0 and b = 800 s/mm^2^ and corresponding apparent diffusion coefficient (ADC) maps of the diffusion phantom obtained using DL-rs-EPI with WEXfs and conventional rs-EPI with SPAIR. (**B**) Comparison of ADC values according to polyvinylpyrrolidone (PVP) concentration. ADC values measured using DL-rs-EPI with WEXfs showed no significant differences compared with those measured using conventional rs-EPI with SPAIR and were comparable to the reference values at 24 °C.

T1-weighted VIBE imaging confirmed a stable phantom temperature of 24 °C throughout the imaging session. Using identical acquisition parameters, the phantom experiment yielded consistent ADC measurements for both conventional rs-EPI and DL-rs-EPI with WEXfs. Representative DWI images and corresponding ADC maps for the two protocols are displayed in Figure 2, demonstrating visually comparable image quality without protocol-specific artifacts.

ADC values derived from b-values of 0, 800, and 1200 s/mm^2^ showed no significant differences between protocols across the range of PVP concentrations when circular ROIs were applied. Across all concentrations, measured ADC values exhibited close agreement with the manufacturer-provided reference values at 24 °C, with deviations ranging from 1.93% to 12.07%. No significant differences in ADC measurements were observed between conventional rs-EPI and DL-rs-EPI with WEXfs (*p* = 0.107, Supplementary Table S1).

### 3.3. Qualitative Analysis and Inter-Reader Agreement

**Figure 3:**
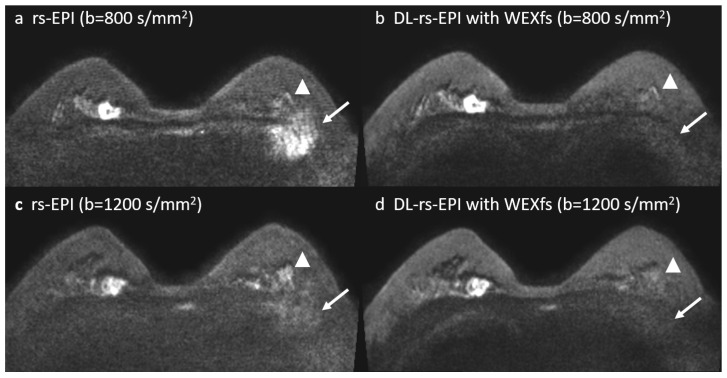
Breast magnetic resonance images of a 46-year-old woman with a 2.6 cm invasive ductal carcinoma (IDC) in the right breast, appearing as a mass lesion. Deep-learning–reconstructed readout-segmented echo-planar imaging (DL-rs-EPI) with water-excitation spectral fat saturation (WEXfs) demonstrated more homogeneous fat suppression with reduced image noise. Conventional rs-EPI diffusion-weighted images displayed pronounced ghosting artifacts along the phase-encoding direction, likely related to shot-to-shot phase inconsistencies and physiologic motion (arrows in (**a**,**c**)). These artifacts are markedly reduced on the DL-rs-EPI with WEXfs images (arrows in (**b**,**d**)). Background diffusion signal anterior to the sternum was reduced on DL-rs-EPI with WEXfs (arrowheads in (**b**,**d**)) compared with rs-EPI (arrowheads in (**a**,**c**)). The ADC value of the IDC was 0.816 × 10^−3^ mm^2^/s on rs-EPI and 0.839 × 10^−3^ mm^2^/s on DL-rs-EPI with WEXfs.

**Figure 4:**
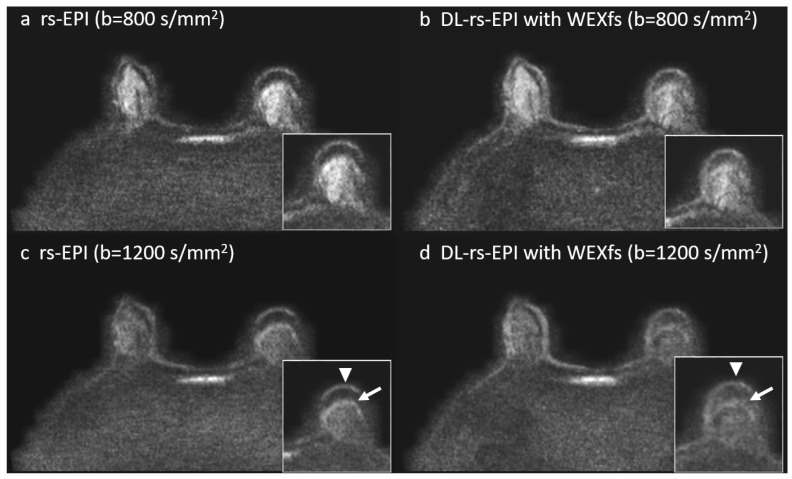
Breast magnetic resonance images of a 58-year-old woman with invasive ductal carcinoma in the right breast. Conventional readout-segmented echo-planar imaging (rs-EPI) images are shown in (**a**,**c**), while deep-learning–reconstructed readout-segmented echo-planar imaging (DL-rs-EPI) with water-excitation spectral fat saturation (WEXfs) images are shown in (**b**,**d**). DL-rs-EPI with WEXfs demonstrates reduced blurring and image noise compared with conventional rs-EPI. At b = 1200 s/mm^2^, susceptibility artifact at the parenchyma–air interface (arrowheads) persists but appears less conspicuous on DL-rs-EPI with WEXfs. Similar distortion is observed at the fat–parenchyma interface (arrows), with improved lesion margin delineation on DL-rs-EPI with WEXfs.

Compared with conventional rs-EPI, DL-rs-EPI with WEXfs demonstrated significantly more homogeneous fat suppression (3.80 ± 0.40 vs. 3.25 ± 0.46, *p* < 0.001) (Table 3, Figure 3) and lower background diffusion signal at both b = 800 s/mm^2^ (2.21 ± 1.08 vs. 2.30 ± 1.06, *p* = 0.008) and b = 1200 s/mm^2^ (1.27 ± 0.57 vs. 1.58 ± 0.76, *p* < 0.001) (Table 3). In a representative case (Figure 3), conventional rs-EPI (panels a and c) revealed pronounced ghosting artifacts along the phase-encoding direction, likely attributable to shot-to-shot phase inconsistencies and physiologic motion; these artifacts were markedly reduced on DL-rs-EPI with WEXfs (panels b and d). Figure 4 illustrates a representative case demonstrating a diminished background diffusion signal on DL-rs-EPI with WEXfs at b = 1200 s/mm^2^. Lesion conspicuity was comparable between the two sequences at b = 800 s/mm^2^ (2.81 ± 0.48 vs. 2.84 ± 0.46, *p* = 0.317) and b = 1200 s/mm^2^ (2.76 ± 0.56 vs. 2.76 ± 0.56, *p* = 1.000). Artifact severity tended to be lower with DL-rs-EPI with WEXfs (0.65 ± 0.78 vs. 0.76 ± 0.90), although this difference was not statistically significant (*p* = 0.088) (Table 3).

Inter-reader agreement for ordinal image quality scores was generally moderate to excellent (Table 4). For the background diffusion signal at b = 800 s/mm^2^, excellent agreement was observed for both DL-rs-EPI with WEXfs (κ = 0.959, 95% CI, 0.935–0.973) and rs-EPI (κ = 0.933, 95% CI, 0.895–0.957). In contrast, lesion conspicuity demonstrated only moderate agreement for both DL-rs-EPI with WEXfs (κ = 0.684, 95% CI: 0.508–0.798) and rs-EPI (κ = 0.556, 95% CI: 0.307–0.715). A similar pattern of inter-reader agreement was observed for DWI acquired at b = 1200 s/mm^2^.

### 3.4. Quantitative Analyses: ROI-Based Relative SNR, CNR, Lesion Contrast, and ADC

Compared with conventional rs-EPI with SPAIR, DL-rs-EPI with WEXfs demonstrated significantly higher ROI-based relative SNR estimates at both b-values of 800 s/mm^2^ (5.79 ± 1.80 vs. 5.28 ± 1.74) and 1200 s/mm^2^ (5.41 ± 1.64 vs. 4.94 ± 1.51; both *p* < 0.001). ROI-based relative CNR estimates were likewise higher with DL-rs-EPI with WEXfs at b = 800 s/mm^2^ (3.35 ± 1.39 vs. 3.12 ± 1.55, *p* = 0.024) and b = 1200 s/mm^2^ (3.67 ± 1.29 vs. 3.37 ± 1.44, *p* = 0.001). Lesion contrast did not differ significantly between the two protocols at either b-value (*p* > 0.1) (Table 5). These SNR and CNR values should be interpreted as ROI-based relative image-quality estimates rather than absolute physical noise measurements, because DL-based reconstruction may alter image noise properties.

With respect to diffusion parameters, lesion ADC values were similar between the two protocols (0.96 ± 0.19 vs. 0.97 ± 0.20 ×10^−3^ mm^2^/s, *p* = 0.084), whereas normal fibroglandular tissue (FGT) ADC values were slightly higher with DL-rs-EPI with WEXfs (1.68 ± 0.28 vs. 1.66 ± 0.26 ×10^−3^ mm^2^/s, *p* = 0.002). Consequently, ADC contrast was significantly greater with DL-rs-EPI with WEXfs (0.73 ± 0.31 vs. 0.69 ± 0.31, *p* < 0.001). Although the increase in normal FGT ADC was statistically significant, the absolute difference was small.

Effect-size analysis showed moderate effects for ROI-based SNR estimates at b = 800 and b = 1200 s/mm^2^ (Cohen’s dz = 0.54 and 0.60, respectively), small effects for CNR estimates at b = 800 and b = 1200 s/mm^2^ (Cohen’s dz = 0.26 and 0.38, respectively), and negligible-to-small effects for lesion contrast and tumor ADC.

## 4. Discussion

In this paired, single-center cohort, a clinically implemented DL-rs-EPI with WEXfs DWI protocol demonstrated higher ROI-based relative SNR and CNR estimates at both b = 800 and b = 1200 s/mm^2^ compared with a conventional rs-EPI protocol, along with improved qualitative image quality and preserved lesion ADC measurements. Although rs-EPI intrinsically reduces geometric distortion compared with single-shot EPI [20], residual noise and phase inconsistencies may persist and may be exacerbated by increasing acceleration factors. Under such challenging high-b-value conditions, reconstruction and fat-suppression strategies that reduce apparent noise, background signal, and phase-related artifacts may provide visible image-quality benefits [21,22,23,24]. These findings are consistent with prior observations that DL-based reconstruction improves image quality, particularly under low-SNR conditions [25,26,27]. However, because the reconstruction method, fat-suppression technique, and acquisition parameters differed simultaneously between the two protocols, the observed image-quality improvements should be interpreted as reflecting the performance of the combined DL-rs-EPI with WEXfs protocol rather than the isolated effect of DL reconstruction alone. Moreover, the ROI-based SNR and CNR values in this study should be regarded as relative image-quality indicators rather than absolute physical noise measurements, because DL reconstruction can alter image noise texture and spatial noise distribution.

Most clinical studies of DL reconstruction have focused on ss-EPI or ss-EPI-based variations incorporating vendor-specific acceleration strategies and have reported higher image-quality metrics, including ROI-based SNR and/or CNR estimates, as well as improved perceived image appearance, primarily through apparent noise reduction while preserving ADC values [9,15,26,27,28]. One study [11] applied DL reconstruction to a multi-shot EPI technique based on MUSE at a single high b-value (b = 800 s/mm^2^), which segments k-space acquisition along the phase-encoding direction, in contrast to rs-EPI, with reported results consistent with observations of previous studies [11]. Moreover, all of these studies employed the SPAIR fat suppression method [9,11,15,26,27]. In this context, the present study provides evidence that a clinically implemented DL-rs-EPI protocol combined with WEXfs yields measurable image-quality enhancement in rs-EPI at both b = 800 and b = 1200 s/mm^2^. Compared with these previous studies, the present study differs in several aspects. First, we evaluated a readout-segmented EPI protocol rather than ss-EPI, allowing assessment of DL reconstruction in a high-resolution multi-shot acquisition. Second, the evaluated protocol incorporated WEXfs instead of the SPAIR fat-suppression method used in most prior breast DWI studies. Third, image quality was assessed at both b = 800 and b = 1200 s/mm^2^, and ADC stability was further supported by phantom validation. These features extend previous evidence by evaluating the clinical feasibility of a combined DL-rs-EPI with WEXfs protocol in breast DWI.

Qualitative analysis in this study demonstrated that DL-rs-EPI with WEXfs provided significant advantages in background diffusion signal (BDS) suppression at both b = 800 s/mm^2^ (2.21 ± 1.08 vs. 2.30 ± 1.06, *p* = 0.008) and b = 1200 s/mm^2^ (1.27 ± 0.57 vs. 1.58 ± 0.76, *p* < 0.001), with a more pronounced effect observed at b = 1200 s/mm^2^. This observation is consistent with fundamental principles of breast DWI, whereby increasing b-values lead to progressive signal attenuation in normal FGT, and the perceived BDS reflects a combination of true diffusion signal and the underlying noise floor [7,29,30,31]. In addition, Stephanie et al. [14] reported that WEXfs improved high-b-value breast DWI image quality compared with conventional fat-suppression approaches, particularly by enhancing background suppression and lesion-to-background contrast. Within this context, the combined effects of DL-based reconstruction, WEXfs, and optimized acquisition parameters may have contributed to lower apparent background signal, reduced perceived noise, and improved high-b-value image quality. Current guidelines recommend a minimum b-value of 800 s/mm^2^ for breast DWI [6] and propose using b-values of 0, 800, and 1200 s/mm^2^ in screening settings [7]. In this framework, acquiring b = 800 s/mm^2^ for quantitative ADC analysis and b = 1200 s/mm^2^ using DL reconstruction with WEXfs to enhance lesion conspicuity and suppress background signal represents a pragmatic screening DWI strategy that improves high-b-value image quality without compromising quantitative integrity.

Lesion ADC values remained unchanged between protocols, suggesting no material bias in tumor diffusivity, consistent with prior reports on DL reconstruction in breast DWI [26,27]. In contrast, normal FGT ADC showed a small but statistically significant increase with DL-rs-EPI with WEXfs in vivo. The absence of significant ADC differences in the phantom experiment supports the interpretation that this small in vivo increase was unlikely to reflect a systematic protocol-related ADC bias under controlled conditions [32,33]. Instead, it may be related to measurement variability in normal FGT, including tissue heterogeneity, intravoxel partial-volume effects, physiologic motion, low parenchymal signal intensity, and DL-related changes in the noise floor [33,34,35,36]. Nevertheless, ADC measurements in low-signal normal FGT should be interpreted with caution, particularly when normal parenchymal ADC, ADC contrast, or lesion-to-background quantitative relationships are used as imaging biomarkers. Although preserved tumor ADC values and comparable lesion conspicuity suggest limited impact on lesion-level interpretation in this study, protocol-specific validation is warranted before normal FGT ADC or ADC contrast is used interchangeably across protocols.

Our qualitative item was designed to capture the homogeneity and adequacy of fat suppression, which directly influences the background signal uniformity on EPI-based breast DWI. As the two protocols employed different fat-suppression strategies (WEXfs vs. SPAIR) and reconstruction methods (DL- vs. non-DL), the superior fat-suppression scores observed with DL–rs-EPI with WEXfs could be attributed to multiple factors. In DL-ss-EPI studies [8,9], no significant differences in homogeneous fat suppression have been reported; however, more homogeneous fat suppression can influence background signal uniformity and ROI-based relative image-quality metrics, including apparent lesion-to-background contrast [37,38]. Accordingly, the observed improvements in fat suppression and ROI-based relative SNR/CNR estimates should be interpreted as protocol-level findings rather than component-specific effects.

This study has several limitations. First, the reconstruction method, fat-suppression technique, and acquisition parameters, including repetition time (TR), differed simultaneously between the two protocols, precluding attribution of the observed differences to any single factor. Therefore, the improvements in ROI-based relative SNR and CNR estimates and qualitative image quality should be interpreted as the combined effect of the overall protocol rather than the effect of DL reconstruction alone. Because this study was designed as a retrospective clinical comparison of two implemented protocols, additional acquisitions using all possible combinations of reconstruction method, fat-suppression technique, and acquisition parameters could not be performed. Prospective component-wise ablation studies are needed to quantify the independent contributions of DL reconstruction, WEXfs, and acquisition-parameter optimization.

Second, traditional ROI-based SNR and CNR measurements may be biased in DL-reconstructed images because nonlinear denoising can alter image noise texture and spatial noise distribution. Therefore, these values should not be interpreted as absolute physical noise measurements, but rather as ROI-based relative image-quality indicators.

Third, although improved image quality may facilitate clinical interpretation, the present study was not designed as a diagnostic accuracy study. The cohort consisted of patients with pathologically confirmed breast cancer who underwent both DWI protocols and did not include a sufficient number of benign lesions or negative control cases. Therefore, analyses of sensitivity, specificity, and AUC were not methodologically appropriate. Future studies using a dedicated diagnostic performance design, including benign and negative cases, are warranted to determine whether the observed image-quality improvements translate into improved lesion detection, diagnostic confidence, or diagnostic accuracy.

Finally, this was a retrospective, single-center study with a relatively small sample size using a single vendor system, which may limit generalizability. Further prospective multicenter studies under controlled acquisition conditions are warranted.

## 5. Conclusions

In this paired single-center study, a clinically implemented DL-rs-EPI with WEXfs DWI protocol demonstrated improved image quality compared with conventional rs-EPI with SPAIR at 3 T breast MRI, including higher ROI-based SNR and CNR estimates and improved background diffusion signal suppression, particularly at b = 1200 s/mm^2^. Tumor ADC values were preserved, and phantom validation showed no significant ADC differences between protocols, supporting the quantitative stability of lesion ADC measurements with the evaluated protocol. Overall, DL-rs-EPI with WEXfs may be feasible for high-quality breast DWI, particularly for high-b-value imaging, while maintaining tumor ADC measurements. However, these findings should be interpreted as reflecting the performance of the combined protocol rather than the isolated effect of DL reconstruction the because reconstruction method, fat-suppression strategy, and acquisition parameters differed between protocols. Further prospective studies are warranted to confirm its diagnostic impact and generalizability.

## Figures and Tables

**Table 1:** Scan parameters of the two DWI sequences.

Scan Parameters	Conventional rs-EPI	DL-rs-EPI with WEXfs
TR/TE1/TE2 (ms)	5760/61/100	5000/61/100
Slice thickness (mm)/Number of slices/Slice gap (mm)	3/50/0	3/50/0
FOV (mm^2^)	320 × 192	320 × 192
Matrix size	220 × 220	220 × 220
In-plane resolution (mm^2^)	0.7 × 0.7	0.7 × 0.7
Acceleration factor	2 (SMS), 2 (GRAPPA)	2 (SMS), 2 (GRAPPA)
Readout partial Fourier	7/8	7/8
Readout segments	5	5
*b*-value (s/mm^2^)	0, 800, 1200	0, 800, 1200
NEX per b-value	1, 1, 2	1, 1, 2
Receiver bandwidth (Hz/px)	947	947
Fat suppression	SPAIR	WEXfs
Acquisition time (min: s)	04:35	04:00

rs-EPI, readout-segmented echo-planar imaging; DL-rs-EPI, deep learning-based reconstruction applied to rs-EPI; TR, repetition time; TE1/TE2, first/second echo time; FOV, field of view; in-plane resolution, pixel size; SMS, simultaneous multi-slice; GRAPPA, Generalized Autocalibrating Partially Parallel Acquisition; readout partial Fourier, fraction of k-space sampled in readout; readout segments, number of readout shots; NEX (NSA), number of excitations/averages per b-value; Hz/px, Hz per pixel; SPAIR, spectral attenuated inversion recovery; WEXfs, water excitation spectral fat saturation method.

**Table 2:** Patient and lesion characteristics.

Characteristics	Value
Number of patients	80
Age (y) *	55 ± 11 (age range, 25–82)
History of breast cancer	
Absent	80 (100.0)
Present	0 (0.0)
Menopausal status	
Pre-menopause	30 (37.5)
Post-menopause	50 (62.5)
Histology of malignant lesion	
Invasive carcinoma, NOS	55 (68.8)
Invasive carcinoma, other specific type ‡	9 (11.3)
DCIS	16 (20.0)
Histologic grade of invasive carcinoma (N = 58) †	
1	11 (19.0)
2	31 (53.4)
3	16 (27.6)
Nuclear grade of DCIS (DCIS N = 16) †	
1	2 (12.5)
2	6 (37.5)
3	8 (50.0)
Predominant lesion on MRI †	
Mass	60 (75.0)
Non-mass	20 (25.0)
Pathologic lesion size, Median (IQR), mm	
All	18.00 (10.5–24.5)
Invasive	15.50 (10.0–23.0)
DCIS	25.00 (13.5–50.5)

NOS, not otherwise specified; DCIS, ductal carcinoma in situ; IQR, interquartile range. * Data are presented as mean ± standard deviations or medians with interquartile ranges in parentheses, as appropriate. † Values are presented as the number of patients with percentages in parentheses. ‡ “Invasive carcinoma, other specific type” included invasive lobular carcinoma (*n* = 5), invasive carcinoma with neuroendocrine differentiation (*n* = 1), invasive carcinoma with micropapillary component (*n* = 1), mixed invasive ductal carcinoma and invasive lobular carcinoma (*n* = 1), and mucinous carcinoma (*n* = 1).

**Table 3:** Results of overall qualitative analysis.

	DL-rs-EPI with WEXfs * (N = 80)	rs-EPI * (N = 80)	*p*-Value
Homogeneous fat suppression	3.80 ± 0.40	3.25 ± 0.46	<0.001
b800: background diffusion signal	2.21 ± 1.08	2.30 ± 1.06	0.008
b1200: background diffusion signal	1.27 ± 0.57	1.58 ± 0.76	<0.001
b800: lesion conspicuity	2.81 ± 0.48	2.84 ± 0.46	0.317
b1200: lesion conspicuity	2.76 ± 0.56	2.76 ± 0.56	1.000
Artifact severity	0.65 ± 0.78	0.76 ± 0.90	0.088

Statistical analysis method: Wilcoxon signed-rank test. * Data represent the mean ± standard deviation of the mean qualitative scores of two readers. rs-EPI, conventional simultaneous multi-slice readout-segmented echo-planar imaging with spectral attenuated inversion recovery; DL-rs-EPI with WEXfs, simultaneous multi-slice readout-segmented echo-planar imaging using deep learning-based reconstruction and water-excitation spectral fat saturation.

**Table 4:** Weighted kappa statistics for inter-reader agreement of qualitative image analysis.

	DL-rs-EPI with WEXfs *	rs-EPI *
Homogeneous fat suppression	0.743 (0.600–0.835)	0.491 (0.207–0.674)
b800: background diffusion signal	0.959 (0.935–0.973)	0.933 (0.895–0.957)
b1200: background diffusion signal	0.886 (0.822–0.927)	0.920 (0.876–0.949)
b800: lesion conspicuity	0.684 (0.508–0.798)	0.556 (0.307–0.715)
b1200: lesion conspicuity	0.619 (0.406–0.756)	0.575 (0.337–0.727)
Artifact severity	0.764 (0.631–0.848)	0.797 (0.684–0.870)

* Data in parentheses are 95% confidence intervals. rs-EPI, conventional simultaneous multi-slice readout-segmented echo-planar imaging with spectral attenuated inversion recovery; DL-rs-EPI with WEXfs, simultaneous multi-slice readout-segmented echo-planar imaging using deep learning-based reconstruction and water-excitation spectral fat saturation.

**Table 5:** Comparison of quantitative parameters between the DL-rs-EPI with WEXfs protocol and conventional rs-EPI with SPAIR protocol.

	DL-rs-EPI with WEXfs (N = 80)	rs-EPI (N = 80)	Mean Paired Difference	*p*-Value	Cohen’s dz
SNR					
800	5.79 ± 1.80	5.28 ± 1.74	0.51 ± 0.95	<0.001	0.54
1200	5.41 ± 1.64	4.94 ± 1.51	0.47 ± 0.79	<0.001	0.60
CNR					
800	3.35 ± 1.39	3.12 ± 1.55	0.23 ± 0.90	0.024	0.26
1200	3.67 ± 1.29	3.37 ± 1.44	0.30 ± 0.80	0.001	0.38
Lesion contrast					
800	3.29 ± 1.64	3.45 ± 2.30	−0.16 ± 1.37	0.313	−0.11
1200	4.21 ± 1.93	4.31 ± 2.32	−0.10 ± 1.41	0.513	−0.07
ADC values of the lesion	0.96 ± 0.19	0.97 ± 0.20	−0.01 ± 0.06	0.084	−0.19
ADC values of normal FGT	1.68 ± 0.28	1.66 ± 0.26	0.02 ± 0.07	0.002	0.36
ADC contrast	0.73 ± 0.31	0.69 ± 0.31	0.04 ± 0.08	<0.001	0.46

Statistical analysis method: Paired *t*-test. Values are presented as mean ± standard deviation. ADC values are expressed in ×10^−3^ mm^2^/s. Cohen’s dz was calculated as the mean paired difference divided by the standard deviation of the paired differences. Differences were calculated as values from DL-rs-EPI with WEXfs minus those from conventional rs-EPI with SPAIR; therefore, positive values indicate higher values in DL-rs-EPI with WEXfs. rs-EPI, conventional simultaneous multi-slice readout-segmented echo-planar imaging with spectral attenuated inversion recovery; DL-rs-EPI with WEXfs, simultaneous multi-slice readout-segmented echo-planar imaging using deep learning-based reconstruction and water-excitation spectral fat saturation; SNR, signal-to-noise ratio; CNR, contrast-to-noise ratio; FGT, fibroglandular tissue.

## Data Availability

The data presented in this study are not publicly available due to privacy and ethical restrictions involving patient information. De-identified data may be available from the corresponding author upon reasonable request and subject to institutional review board approval.

## Supplementary Materials

The following supporting information can be downloaded at: https://www.mdpi.com/article/10.3390/diagnostics16131958/s1, Table S1: ADC values of phantom measured from two DWI sequences at 24 °C.

## Abbreviations

The following abbreviations are used in this manuscript:

DWI	diffusion-weighted imaging
EPI	echo-planar imaging
DL	deep learning
ss-EPI	single-shot echo-planar imaging
rs-EPI	readout-segmented echo-planar imaging
ADC	apparent diffusion coefficient
SPAIR	spectral attenuated inversion recovery
WEXfs	water excitation spectral fat saturation method
SNR	signal-to-noise ratio
CNR	contrast-to-noise ratio
DCE	dynamic contrast-enhanced
BDS	background diffusion signal
CI	confidence interval
