# Supplementary material for: Clinical Evaluation of a Combined Deep Learning–Reconstructed Readout-Segmented Echo-Planar Imaging and Water-Excitation Spectral Fat-Saturation Protocol for Breast Diffusion-Weighted Imaging at 3T Breast MRI
Source: Diagnostics (Basel). 2026 Jun 24;16(13):1958. doi: 10.3390/diagnostics16131958 (PMC13359702; doi:10.3390/diagnostics16131958)
Supplement: Supplementary file 1 [file diagnostics-16-01958-s001.zip › diagnostics-4358615-supplementary.pdf]

Supplementary Table S1. ADC values of phantom measured from two DWI sequences at 24°C

	Reference (24°C)*	DL-rs-EPI with WEXfs		Conventional rs-EPI with SPAIR		p-value†
		ADC*	Diff. from Ref (%)	ADC*	Diff. from Ref (%)	
Conc. 1 (0%)	2.232 ± 0.048	2.351 ± 0.025	5.332	2.275 ± 0.033	1.927	0.107
Conc. 2 (10%)	1.742 ± 0.038	1.781 ± 0.008	2.239	1.765 ± 0.002	1.378	
Conc. 3 (20%)	1.322 ± 0.029	1.378 ± 0.015	4.085	1.366 ± 0.015	3.328	
Conc. 4 (30%)	0.929 ± 0.022	0.962 ± 0.003	3.552	0.953 ± 0.001	2.583	
Conc. 5 (40%)	0.584 ± 0.015	0.635 ± 0.006	8.733	0.627 ± 0.004	7.363	
Conc. 6 (50%)	0.323 ± 0.009	0.362 ± 0.013	12.074	0.354 ± 0.006	9.598	

Data represent means ± standard deviations from two repeated measurements per vial. * ADC value, 10^{-3} mm^2/s.

Reference values are temperature-compensated using manufacturer tables at T=24°C.

† p-value from paired t-tests comparing conventional rs-EPI with SPAIR and DL-rs-EPI with WEXfs for each concentration. rs-EPI, conventional simultaneous multi-slice readout-segmented echo-planar imaging with spectral at-tenuated inversion recovery; DL-rs-EPI with WEXfs, simultaneous multi-slice readout-segmented echo-planar imaging using deep learning–based reconstruction and water-excitation spectral fat saturation.

Glossary

ADC (Apparent Diffusion Coefficient): Measure of water diffusion within tissue ($\times 10^{-3}$ mm^2/s).

b-value: Parameter controlling diffusion weighting in DWI.

Breast MRI: Imaging of breast tissue using magnetic resonance techniques.

Calibrated Diffusion Phantom: Standardized device for validating ADC measurements.

CNR (Contrast-to-Noise Ratio): Signal difference between lesion and tissue relative to noise.

DLR (Deep Learning Reconstruction): Neural network–based method to enhance MRI image quality.

DWI (Diffusion-Weighted Imaging): MRI sensitive to water molecule diffusion in tissue.

EPI (Echo-Planar Imaging): Fast MRI acquisition method, prone to distortion and artifacts.

Fat Suppression: Technique to reduce fat signal and improve lesion visibility.

rs-EPI (Readout-Segmented EPI): Multi-shot EPI reducing distortion and blurring.

ROI (Region of Interest): Area selected for quantitative analysis on an image.

SNR (Signal-to-Noise Ratio): Ratio of image signal to background noise.

SMS (Simultaneous Multi-Slice Acceleration): Acquires multiple slices simultaneously to shorten scan time.

ss-EPI (Single-Shot EPI): EPI acquiring all k-space lines in one excitation.

BDS (Background Diffusion Signal): Residual signal in normal tissue on DWI.

Weighted Kappa (κ): Statistic measuring inter-rater agreement for ordinal data.

DCE MRI (Dynamic Contrast-Enhanced MRI): Uses contrast to evaluate tissue vascularity.

FGT (Fibroglandular Tissue): Normal fibrous and glandular breast tissue.
